# Deformity of the Mouth—Dieffenbach's Operation

**Published:** 1870-11

**Authors:** 


### ARTICLE VIII.

### *Deformity of the Mouth—Dieffenbach's Operation.*

The next case which I have to show you is Robert C., æt. 11 years; suffering from a deformity of the mouth, resulting from the contraction of the cicatrix of a slough, which slough was one of the many concomitants of scarlatina, a disease which, as you know, may be followed by lesion of almost any structure or portion of the body.

You will perceive the left angle of the mouth is drawn downward and pulled in, or puckered in such a manner as to give an unpleasant appearance to his face, and he desires us to rectify this condition. This is not a common condition, yet it is sometimes met with from wounds, bruises, etc., and we must study the complications which exist in each case, governing our operation by the extent and character of the contraction.

In looking at a case of this kind you must bring to bear upon it all your artistic as well as surgical skill; you must look at and view it in different positions, until you have decided what alterations are necessary to bring this deformed mouth into a normal condition, with the labial curves and commissures in their usual beautiful symmetry; and, in order to do this, map out in your mind, or, still better, in ink upon the face, the proper outlines of a perfect mouth; then you can arrange your incisions to suit the particular deformity.

In the present instance the contraction is not so great as is often seen, where the orifice is sometimes so small as scarcely to admit of the passage of the little finger, and it is, therefore, comparatively an easy case.

For the relief of this difficulty, our first thought would be to merely incise the commissures to the required extent; but unfortunately such a procedure would only be followed by speedy union and greater consequent contraction than before since it is impossible to prevent union of the fresh surfaces, even though tents, lead, ect., be placed between the edges.

Mechanical dilatation gives severe pain and does not produce any permanent good.

The only method which has proved of service has been that of Dieffenbach, of Berlin, which is performed by the surgeon first introducing the fingers of one hand into the mouth of the patient, in order to guide and direct the incisions and make the cheek tense, during which time he should stand either behind or in front of his patient, according to the side of the mouth upon which he is to operate, and then inserting the blade of a pair of scissors into the cheek about two lines outside the point which he intends shall be the labial commissure, he cuts inward and upward, dividing all the tissues save the mucous membrane, until he has reached a point in the upper lip which will when brought down, fulfill the required conditions. Again, a similar incision is made from the same starting point, but this time, toward the lower lip which, in turn, is divided nearly to the same extent as above; the cuts in each instance being varied to suit the irregularities in the position of the contracted orifice. Next dissect away the flesh down to the submucous coat, thus leaving but the mucous membrane, which must in its turn be divided by a straight line running out its centre, to a point two lines inside the angle of external incision.

The mucous bembrane is now turned over to imitate the normal lip, above and below on each side, and, its edges sutured to those of the skin, thus forming the vermillion border of a new lip.

This operation usually answers admirably, but sometimes unfortunately, the mucous membrane also participates in the lesion, and the operation cannot be successfully performed; or again, certain accidents may come between the surgeon and success, and cases will be found where this operation will not cure.

In such cases I am in the habit of modifying, or rather of adding to, or associating a mechanical appliance with these surgical means.

This consists of what might be called a "mouth stretcher," an instrument made of wood or rubber, with a deep groove around its border, which is slipped between the lips after time has been given for union of the reflected mucous membrane. The lips will be caught and held by the gutter of the apparatus, in the whole of their circumferences, and thus not only is the healing influenced to a desired shape, but undue cicatrical contraction is also prevented. This instrument should be worn constantly for a week or two, after which time its use should be continued at night until all contraction has ceased. This apparatus also does more than merely prevent contraction; it may even remedy defects which are the fault of the operator, by compelling the regular healing of the wound, and such mistakes and blunders you will make unless you carefully weigh each cut, and look at it in various aspects—you must bring all your mechanico-surgico skill into use.

[The incisions were then made as described, the tissues being removed down to the mucous membrane, which was brought over and firmly stitched to the integument. At each step of the operation the case was viewed both from a near and distant standpoint, thus rendering the final adjustment a more perfect success. The hemorrhage was but slight, and soon ceased. Cold water dressings were applied, and the boy directed not to exert and strain upon his mouth until union should be well accomplished.—Dr. F. W.] Clinic of Professor Garretson, University of Penn.—*Medical & Surgical Reporter.*

